# Evaluating the Use of a Note-Taking App by Japanese Resident Physicians: Nationwide Cross-Sectional Study

**DOI:** 10.2196/55087

**Published:** 2025-05-09

**Authors:** Taiju Miyagami, Yuji Nishizaki, Taro Shimizu, Yu Yamamoto, Kiyoshi Shikino, Koshi Kataoka, Masanori Nojima, Gautam A Deshpande, Toshio Naito, Yasuharu Tokuda

**Affiliations:** 1 Department of General Medicine, Faculty of Medicine, Juntendo University, Bunkyo-ku Hongo 3-1-3, Tokyo, 113-8421, Japan, 81 338133111; 2 Division of Medical Education, School of Medicine, Juntendo University, Tokyo, Japan; 3 Department of Diagnostic and Generalist Medicine, Dokkyo Medical University Hospital, Tochigi, Japan; 4 Division of General Medicine, Center for Community Medicine, Jichi Medical University, Tochigi, Japan; 5 Department of General Medicine, Chiba University Hospital, Chiba, Japan; 6 Department of Community-Oriented Medical Education, Chiba University Graduate School of Medicine, Chiba, Japan; 7 Center for Translational Research, The Institute of Medical Science, The University of Tokyo, Tokyo, Japan; 8 Muribushi Okinawa Center for Teaching Hospitals, Okinawa, Japan; 9 Tokyo Foundation for Policy Research, Tokyo, Japan

**Keywords:** note-taking, applications, digital notes, resident physicians, medical education

## Abstract

**Background:**

Note-taking is a method that has long been used to optimize studying. Recent innovations have seen the introduction of digital note-taking using software apps. Although the current state of digital note-taking has been verified mainly among students, the use and efficacy of digital note-taking by physicians in actual clinical practice remain unknown. Therefore, we sought to understand the characteristics of note-taking residents using a note-taking app and determine whether there is a difference in basic medical knowledge compared to that of nondigital note-taking residents.

**Objective:**

This study investigated the use of a digital note-taking app by Japanese resident physicians.

**Methods:**

This analytical cross-sectional study was conducted in resident physicians during the General Medicine In-Training Examination (GM-ITE), a clinical competency examination for resident physicians. The GM-ITE is a multiple-choice test with a maximum score of 80 points. Using a structured questionnaire, we collected data on the sociodemographic characteristics (sex, age, postgraduate year [PGY], or others), clinical training, GM-ITE scores, and the use of an app for note-taking to record case experience. The GM-ITE evaluated the scores by dividing them into 4 groups (groups 1‐4), in order from the lowest to the highest. We conducted a multivariate analysis of sociodemographic, clinical training, and GM-ITE score variables to determine the independent predictors of the use of a digital note-taking app.

**Results:**

This study included 3833 participants; 1242 (32.4%) were female, 1988 (51.8%) were PGY 1 residents, 2628 (68.6%) were training in a rural area, 3236 (84.4%) were in community-based hospitals, and 1750 (45.3%) were app users. The app users were more likely to be in their PGY 2, to work in a community-based hospital, to have general internal medicine rotation experience, to use online medical resources more frequently, and to have more time for self-study. The results showed that the app users group had a higher GM-ITE score than the nonapp users group (adjusted odds ratio 0.74, 95% CI 0.25 to 1.22; *P*=.003).

**Conclusions:**

To the best of our knowledge, this is the first study to investigate note-taking by physicians in Japan using apps. The app users group had a higher GM-ITE score than the nonapp users, suggesting that they may have higher clinical skills. In the future, we would like to conduct more in-depth research on the facts of note-taking using apps, based on our results.

## Introduction

Note-taking during learning has long been a mainstay of educational practice and data over the last 60 years, demonstrating its contribution to improved learning and test scores [1,2]. In 1995, a study on effective note-taking among students reported that free note-taking by learners was a particularly effective process [3]. A 2002 report noted that the most effective way for medical students to perform well was to take written notes on materials prepared in advance by the teachers [4]. Since 2010, the use of mobile technology in education has increased in medical and pharmacy schools and is correlated with high student satisfaction [5,6]. Note-taking is also useful to improve understanding during the class and to retrospectively reflect on learned material [7].

In recent years, medical students have been using digital devices extensively for learning and literature searches [8]. Moreover, in medical education, digital methods are often used mainly as a replacement for traditional methods [9]. These days, medical students have increasingly encouraged incorporating digital tools, such as the iPad, for note-taking [10]. It has been suggested that digital note-taking may be better than nondigital note-taking concerning readability and search capabilities during revision of the notes [11]. Furthermore, students tend to have a positive attitude toward learning the use of digital tools [12]. They are reportedly able to use mobile devices as online information resources, which leads to improved learning efficiency in the classroom [13]. Multiple mobile devices are also being used in clinical settings. They are used for telemedicine, and have been developed to support clinical decision-making, providing timely feedback to residents in addition to improving clinical skills [14,15].

However, there are also disadvantages to the use of digital tools such as apps. One of the disadvantages of using apps in clinical settings is the challenge of using them well in multiple electronic tools [8]. First, medical students are not used to digital technology, and a tendency exists for them to prefer paper-based materials to digital ones [5], which is supported by a study that reported the digital group performing worse than the group that took notes using paper and pen [16]. Multitasking may have been a factor in the poor performance [16]. Additionally, a study in Mexico has also raised the issue of the difficulty of using online content when the internet connection is poor, and whether or not the instructor is willing to allow the use of online content [17].

However, we are unaware of the type of individuals using a note-taking app and the background factors, such as the extent to which clinical skills differ between app users and nonapp users.

Therefore, we sought to understand the characteristics of note-taking residents using a note-taking app and determine whether there is a difference in the basic medical knowledge compared to that of nondigital note-taking residents.

## Methods

### Design and Participants

This was a nationwide analytical cross-sectional survey of resident physicians in Japan. Since 2004 in Japan, newly graduated resident physicians have undergone mandatory cross-disciplinary 2-year training (including rotations in internal medicine, emergency medicine, pediatrics, gynecology, psychiatry, surgery, and community medicine) after passing the national medical examination during their final year of medical school [18]. The departments for rotational training and duration and timing during the 2 years of training vary from hospital to hospital and are not set in stone. Resident physicians in their first (postgraduate year [PGY] 1) and second (PGY 2) years take the same General Medicine In-Training Examination (GM-ITE), and examination participants complete questionnaires that survey the actual status of resident physicians immediately after the examination. Participants were gathered by an announcement from the training supervisor at each teaching hospital, and resident physicians participated voluntarily. This study was conducted between January 17, 2023, and January 30, 2023, and followed STROBE (Strengthening the Reporting of Observational Studies in Epidemiology) guidelines [19].

### Variables

The GM-ITE assesses general clinical knowledge and its practical relevance by the medical training guidelines of the Ministry of Health, Labor, and Welfare (MHLW) of Japan. This exam provides feedback to individual resident physicians and evaluates training programs and facilities. The GM-ITE comprises 80 multiple-choice questions, with optional tests that are answered by approximately 50% of all resident physicians in Japan. The GM-ITE is a test organized by the Japan Association for Medical Education Program (JAMEP). JAMEP is a nonprofit organization established with the aim of contributing to the promotion of Japanese medicine through support for training and education to improve the quality of medical care, as a third-party organization that checks the quality of medical education in Japan. The authors received the data from the person in charge at JAMEP. In line with the MHLW’s goals for resident physicians, the 2022 GM-ITE consisted of a total of 80 structured questions, including (1) eight questions on medical history taking and professionalism, (2) eighteen questions on symptomatology and clinical reasoning, (3) eighteen questions on physical examination and clinical skills, and (4) thirty-six questions on basic clinical knowledge, including disease knowledge. The GM-ITE is answered on the computer, and after the test is finished, the participant decides whether or not to agree to participate in questionnaires on the computer. Thereafter, they answer the following questions. Baseline characteristics included resident physicians’ age, gender, year in training, and hospital location (urban or rural; the 20 cities designated by the Ministry of Internal Affairs and Communications and the 23 wards of Tokyo were defined as urban areas, and all other cities were defined as rural areas), general hospital type (community-based or university hospital), number of beds, whether rotation in a general medicine department was completed, number of emergency department shifts per month, average number of inpatients cared for daily, use of medical online resources, self-study time per day, and duty hours per week. The ages of the resident physicians were divided into 3 groups. Group 1 consisted of resident physicians aged 24 and 25 years, group 2 consisted of physicians aged 26 and 27 years, and group 3 consisted of physicians aged 28, 29, and 30 years and older. The main outcome question regarding app use was asked as follows. First, participants were asked whether they kept records of the cases they had experienced, and those who did not were excluded. They were then asked whether they used the application software to record the cases they had experienced, and they responded with either yes or no. The relationship between the total GM-ITE score and app use was also investigated. The GM-ITE is taken by residents under supervision at hospitals. However, due to the recent COVID-19 pandemic, some residents took the examination from their homes. The data of residents who took the exam at home were excluded from the analysis. Participants who did not collect cases for self-study, either on paper or through an app, were excluded. Additionally, those with missing data were excluded.

### Ethical Considerations

This study was approved by the JAMEP Ethics Committee (No. 22‐31). The study was conducted in accordance with the principles of the Declaration of Helsinki [20]. All the methods were performed following the Ethical Guidelines for Medical and Health Research Involving Human Subjects.

All participants reviewed this study document detailing data anonymization, voluntariness, and the dissemination of research outcomes before involvement. Only participants who provided informed consent were included in this study. The data are being carefully managed by the JAMEP staff in a locked file. The data were anonymized at the time of passing them to the authors; thus, it is not possible to identify the participants. In particular, the questions in this study are limited to minor invasive questions. The participants answered the questions on a completely voluntary basis, and no compensation, including rewards, was provided.

### Data Analysis

The results are presented as means (SD) for continuous variables or as prevalence (%) for categorical variables. During data analysis, the subgroup analysis (app users vs app nonusers) were performed to determine the factors associated with the app use. Comparisons between the 2 groups were performed using the chi-square test. Univariate or multivariate analyses with a mixed logistic regression model were performed using a note-taking app to collect cases as the objective variable.

Other fixed covariates were sex, age, PGY, hospital location, hospital type, bed number, general medicine department rotation, number of emergency department duties per month, average number of inpatients cared for daily, use of medical online resources, self-study time per day, and duty hours per week. The GM-ITE scores were divided into 4 quartiles from top to bottom. Group 1 had the lowest score groups and group 4 had the highest score.

The total GM-ITE score was used as the objective variable for the analysis of secondary outcomes. Univariate or multivariate analyses with a linear mixed model were conducted using the GM-ITE total score as the objective variable, with a random effect of the training hospital. The fixed covariate was the presence or absence of app use, in addition to the previously mentioned variables.

The 95% CIs were defined using the mixed logistic regression and linear mixed models. All calculations were performed using SAS (version 9.4; SAS Institute). *P* values <.05 were considered statistically significant. The sample size was not calculated for this exploratory study.

## Results

**Figure 1.**
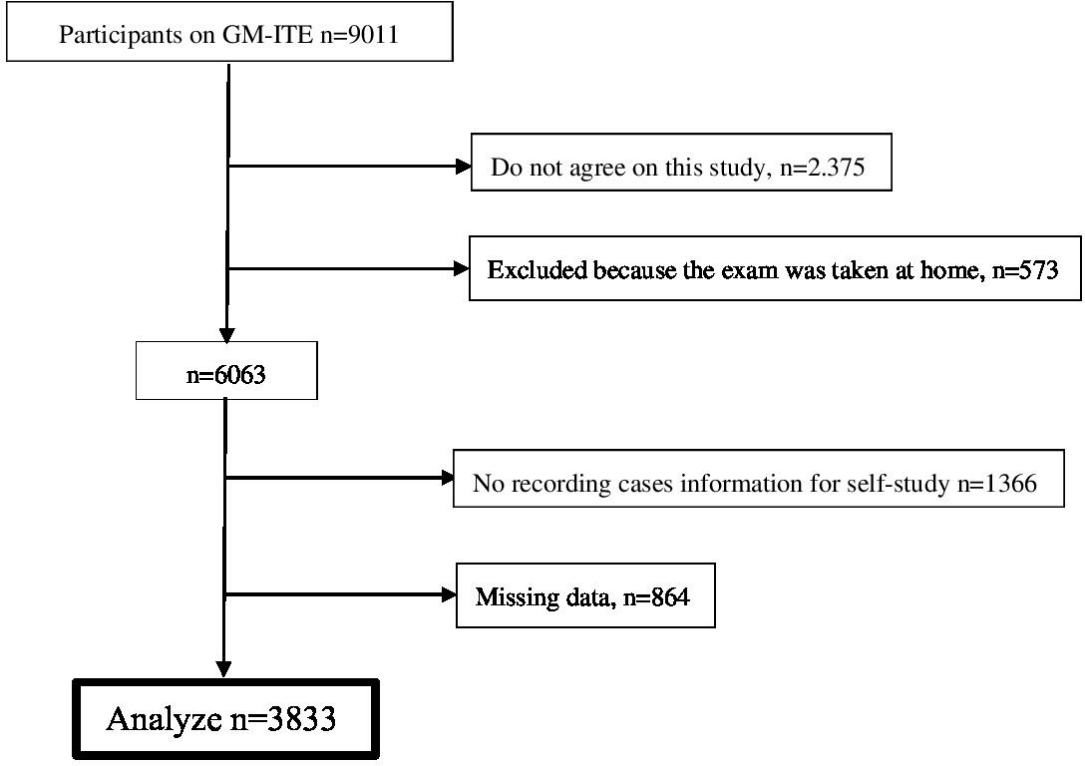
Participant characteristics. GM-ITE: General Medicine In-Training Examination.

A total of 9011 residents from 662 teaching hospitals participated in the 2022 GM-ITE. Participants who did not provide consent (n=2375), took the GM-ITE at home (n=573), did not record their cases for self-study purposes (n=1366), and had at least 1 missing data variable (n=864) were excluded. A total of 3833 participants were included in the final analysis (Figure 1).

Most (n=2591, 67.6%) of the participants were female, 32.4% (n=1242) were PGY-1 resident physicians, and 52.4% (n=2009) were of age group 2. Most (n=2628, 68.6%) of them were training in rural areas, and 84.4% (3236) practiced in community-based hospitals, while less than half (n=1772, 46.2%) had completed a general medicine rotation. Emergency department duty: most (n=2766, 72.2%) of the participants had 3‐5 duties per month in the emergency department. Almost half (n=1896, 49.5%) of the participants had less than 60 duty hours per week. Nearly half (n=1933, 50.4%) reported caring for 5‐9 inpatients daily. Furthermore, less than one-third of the participants (n=1095, 28.6%) were using online medical resources, while 41.4% (n=1586) and 41% (n=1570) spent 0‐30 minutes and 31‐60 minutes for self-study time per day, respectively (Table 1). The participants’ GM-ITE scores ranged from 12 to 71 points.

A substantial proportion (n=1750, 45.3%) of participants were using the note-taking app.

Among this study’s participants, PGY-2 residents used the app more frequently than PGY-1 residents (n=896, 48.6% vs n=843, 42.4%, respectively; adjusted odds ratio [aOR] 1.10, 95% CI 1.03 to 1.41; *P*=.02). Participants in community-based hospitals were more likely to use the app than those in university hospitals (n=1513, 46.8% vs n=226, 37.9%, aOR 1.42, 95% CI 1.04 to 1.94; *P*=.03). Those who worked in hospitals with a larger number of beds were also more likely to use the app (aOR 1.08, 95% CI 1.02 to 1.13; *P*=.004). Those who had rotated through the Department of General Medicine were more likely to use the app than those who had not (n=881, 49.7% vs n=858, 41.6%, aOR 1.19, 95% CI 1.02 to 1.39; *P*=.03). The number of inpatients cared for per day was most frequently 10‐14 among app users, which showed a significant difference (aOR 1.62, 95% CI 1.18 to 2.21; *P*=.003). Participants in the app user group also used online medical resources more frequently (n=1365, 49.9% vs n=374, 34.2%, aOR 1.52, 95% CI 1.3 to 1.79; *P*<.001; Table 2).

The GM-ITE total score was used for evaluation. The following variables showed significant differences in the GM-ITE total score in the multivariate analysis. Female scores tended to be lower than male scores (adjusted difference [aD] −1.18, 95% CI −1.69 to −0.68; *P*<.001). PGY-2 scores tended to be higher than PGY-1 scores (aD 2.45, 95% CI 1.92 to 2.99; *P*<.001). The age of the resident physician tended to be lower in both group 2 and group 3 than in group 1, with lower GM-ITE scores (group 2 [aD −2.55, 95% CI −3.17 to −1.92; *P*<.001] and group 3 [aD -5.88, 95%CI −6.62 to −5.13; *P*<.001]). Predictors of the high GM-ITE scores were working at community-based hospitals (aD 3.64, 95% CI 2.44 to 4.84; *P*<.001), hospitals with more beds (aD 0.36, 95% CI 0.17 to 0.55; *P*<.001), high number of emergency department duties per month (aD 1.98, 95% CI 0.15 to 3.80; *P*=.03), high number of inpatient (aD 1.40, 95% CI 0.32 to 2.47; *P*=.01), using medical online resources (aD 1.45, 95% CI 0.9 to 1.99; *P*<.001), and high number of self-study time (aD 1.41, 95% CI 0.13 to 2.69; *P*=.03). The GM-ITE score tended to be higher for the group working 60 to 79 hours per week than for the group working 0 to 59 hours per week (aD 0.97, 95% CI 0.45 to 1.59; *P*<.001). However, for the group working 80 hours or more per week, there was no difference in score from the group working 0 to 59 hours per week, suggesting that working 60 to 79 hours per week is optimal for improving the GM-ITE score. Moreover, app users had significantly higher scores (aD 0.74, 95% CI 0.25 to 1.22; *P*=.003; Table 3).

**Table 1.** Participant characteristics (N=3833).

Participants’ profile	Total
Sex, n (%)	
Female	1242 (32.4)
Male	2591 (67.6)
Grade, n (%)	
PGY-1^a^	1.8 (51.9)
PGY-2	1845 (48.1)
Age, n (%)	
Group 1	891 (23.3)
Group 2	2009 (52.4)
Group 3	933 (24.3)
Hospital location, n (%)	
Urban	1205 (31.4)
Rural	2628 (68.6)
Hospital type	
Community-based hospital, n (%)	3236 (84.4)
University hospital, n (%)	597 (15.6)
Bed number, mean (SD)	547 (220)
General medicine department rotation, n (%)	
Yes	1772 (46.2)
No	2061 (53.7)
Emergency department duty, n (%)	
0 per month	94 (2.5)
1‐2 per month	615 (16)
3‐5 per month	2766 (72.2)
>6 per month	347 (9.1)
Unknown	11 (0.3)
Number of cared for inpatients, n (%)	
0‐4	1481 (38.6)
5‐9	1933 (50.4)
10‐14	260 (6.7)
15 and over	76 (2)
Unknown	83 (2.2)
Using medical online resources, n (%)	
Yes	1095 (28.6)
No	2738 (71.4)
Self-study time per day, n (%)	
0‐30 minutes	1586 (41.4)
31‐60 minutes	1570 (41)
61‐90 minutes	482 (12.6)
91 minutes or more	143 (3.7)
None	52 (1.4)
Duty hour (h/wk), n (%)	
0‐59	1896 (49.5)
60‐79	1401 (36.6)
>80	536 (14)
GM-ITE^b^ score total, mean (SD)	45.9 (8.2)
Group 1, range	12‐40
Group 2, range	41‐46
Group 3, range	47‐52
Group 4, range	53‐71

a PGY: postgraduate year.

b GM-ITE: General Medicine In-Training Examination.

**Table 2.** Comparison of app-using and nonusing groups. We conducted a logistic regression analysis comparing people who used the app with those who did not, using those who did not use the app as the reference.

		App use (n=1750), n (%)	Nonapp use (n=2133), n (%)	OR^a^	Univariable (95% CI)	*P* value	aOR^b^	Multivariable (95% CI)	*P* value
Gender									
	Male	1175 (45.3)	1416 (54.7)	Reference	N/A^c^	N/A	N/A	N/A	N/A
	Female	564 (45.4)	678 (54.6)	1	0.88 to 1.15	.96	1.10	0.95 to 1.28	.20
Grade									
	PGY-1^d^	843 (42.4)	1145 (57.6)	Reference	N/A	N/A	N/A	N/A	N/A
	PGY-2	896 (48.6)	949 (51.4)	1.28	1.13 to 1.46	<.001	1.20	1.03 to 1.41	.02
Resident age									
	Group 1	407 (45.7)	484 (54.3)	Reference	N/A	N/A	N/A	N/A	N/A
	Group 2	944 (47)	1065 (53)	1.05	0.9 to 1.24	.51	0.97	0.8 to 1.16	.71
	Group 3	388 (41.6)	545 (58.4)	0.85	0.7 to 1.02	.08	0.88	0.7 to 1.1	.27
Hospital location									
	Urban	545 (45.2)	660 (54.8)	Reference	N/A	N/A	N/A	N/A	N/A
	Rural	1194 (45.4)	1434 (54.6)	1.01	0.88 to 1.16	.85	1.03	0.85 to 1.24	.80
Hospital type									
	University Hospital	226 (37.9)	371 (62.1)	Reference	N/A	N/A	N/A	N/A	N/A
	Community-based hospital	1513 (46.8)	1723 (53.2)	1.42	1.21 to 1.72	<.001	1.42	1.04 to 1.94	.03
Bed number		555 (219)^e^	539 (221)^e^	1.03	1 to 1.06	.02	1.08	1.02 to 1.13	.004
General medicine department rotation									
	No	858 (41.6)	1203 (58.4)	Reference	N/A	N/A	N/A	N/A	N/A
	Yes	881 (49.7)	891 (50.3)	1.37	1.21 to 1.58	<.001	1.19	1.02 to 1.39	.03
Emergency department duty									
	0 per month	32 (34)	62 (66)	Reference	N/A	N/A	N/A	N/A	N/A
	1‐2 per month	250 (40.7)	365 (59.3)	1.33	0.84 to 2.1	.22	1.16	0.71 to 1.91	.56
	3‐5 per month	1276 (46.1)	1490 (53.9)	1.66	1.08 to 2.56	.02	1.32	0.82 to 2.14	.26
	>6 per month	177 (51)	170 (49)	2.02	1.25 to 3.25	.004	1.6	0.93 to 2.75	.09
	Unknown	4 (36.3)	7 (63.6)	1.11	0.3 to 4.06	.88	0.81	0.2 to 3.29	.77
Number of cared for inpatients									
	0‐4	623 (42.1)	858 (57.9)	Reference	N/A	N/A	N/A	N/A	N/A
	5‐9	903 (46.7)	1030 (53.3)	1.21	1.05 to 1.38	.007	1.03	0.88 to 1.21	.70
	10‐14	149 (59.6)	111 (40.4)	1.85	1.42 to 2.41	<.001	1.62	1.18 to 2.21	.003
	15 and over	33 (43.4)	43 (56.6)	1.06	0.66 to 1.68	.82	0.92	0.54 to 1.56	.74
	Unknown	31 (42.5)	52 (57.5)	0.82	0.52 to 1.30	.41	0.77	0.47 to 1.26	.30
Using medical online resources									
	No	374 (34.2)	721 (65.8)	Reference	N/A	N/A	N/A	N/A	N/A
	Yes	1365 (49.9)	1373 (50.2)	1.92	1.66 to 2.22	<.001	1.52	1.3 to 1.79	<.001
Self-study time per day									
	0‐30 min	625 (39.4)	961 (60.6)	Reference	N/A	N/A	N/A	N/A	N/A
	31‐60 min	743 (47.3)	827 (52.7)	1.38	1.2 to 1.59	<.001	1.23	1.06 to 1.44	.008
	61‐90 min	275 (57.1)	207 (42.9)	2.04	1.66 to 2.51	<.001	1.79	1.43 to 2.24	<.001
	91 min or more	82 (57.3)	61 (42.7)	2.07	1.46 to 2.92	<.001	1.63	1.12 to 2.37	.01
	None	14 (26.9)	38 (73.1)	0.57	0.31 to 1.06	.07	0.59	0.31 to 1.14	.12
Duty hour (h/wk)									
	0‐59	803 (42.4)	1093 (57.6)	Reference	N/A	N/A	N/A	N/A	N/A
	60‐79	661 (47.2)	740 (52.8)	1.22	1.06 to 1.4	.006	0.99	0.85 to 1.15	.88
	>80	275 (51.3)	261 (48.7)	1.43	1.18 to 1.74	<.001	1.12	0.9 to 1.39	.31
GM-ITE^f^ score									
	Group 1^g^	370 (37.8)	609 (62.2)	Reference	N/A	N/A	N/A	N/A	N/A
	Group 2	375 (42.1)	515 (57.9)	1.2	1 to 1.44	.06	1.06	0.87 to 1.29	.58
	Group 3	449 (45.9)	529 (54.1)	1.4	1.17 to 1.67	<.001	1.15	0.94 to 1.40	.18
	Group 4	545 (55.3)	441 (44.7)	2.03	1.7 to 2.44	<.001	1.42	1.15 to 1.75	.001

a OR: odds ratio.

b aOR: adjusted odds ratio.

c N/A: not applicable.

d PGY: postgraduate year.

e Mean (SD).

f GM-ITE: General Medicine In-Training Examination.

g The GM-ITE group was classified into four quartiles in order of score, from group 1.

**Table 3.** Evaluation based on total GM-ITE^a^ score. Analysis using multiple regression analysis.

		Difference	Univariable (95% CI)	*P* value	Adjusted difference	Multivariable (95% CI)	*P* value
Gender							
	Female (vs male)	−0.86	−1.41 to −0.31	.002	−1.18	−1.69 to −0.68	<.001
Grade							
	PGY-2^b^ (vs PGY-1)	1.4	0.88 to 1.92	<.001	0.18	−0.04 to 0.4	.11
Resident age							
	Group 1	N/A^c^	N/A	N/A	N/A	N/A	N/A
	Group 2	−1.38	−2.01 to −0.75	<.001	−2.55	−3.17 to −1.92	<.001
	Group 3	−5.29	-6.02 to −4.55	<.001	−5.88	−6.62 to −5.13	<.001
Hospital location							
	Rural (vs urban)	0.06	−0.5 to 0.63	.82	0.49	−0.25 to 1.22	.19
Hospital type							
	Community-based hospital (vs university hospital)	4.58	3.88 to 5.28	<.001	3.64	2.44 to 4.84	<.001
Bed number		−0.07	−0.19 to 0.05	.28	0.36	0.17 to 0.55	<.001
General medicine department rotation		1.5	0.98 to 2.02	<.001	0.6	0.06 to 1.15	.03
Emergency department duty							
	0 per month	N/A	N/A	N/A	N/A	N/A	N/A
	1‐2 per month	1.72	−0.04 to 3.5	.06	0.33	−1.32 to 1.99	.69
	3‐5 per month	4.18	2.51 to 5.86	<.001	1.23	−0.38 to 2.83	.13
	>6per month	5.17	3.31 to 7.03	<.001	1.98	0.15 to 3.8	.03
	Unknown	3.1	−1.99 to 8.19	.23	2.45	−2.25 to 7.15	.31
Number of cared for inpatients							
	0‐4	N/A	N/A	N/A	N/A	N/A	N/A
	5‐9	2.09	1.54 to 2.64	<.001	1.48	0.95 to 2.02	<.001
	10‐14	2.06	1 to 3.14	<.001	1.4	0.32 to 2.47	.01
	15 and over	1.53	−0.35 to 3.41	.11	0.51	−1.32 to 2.34	.59
	Unknown	0.57	−1.24 to 2.37	.54	0.31	−1.33 to 1.95	.71
Using medical online resources		3.11	2.9 to 3.68	<.001	1.45	0.9 to 1.99	<.001
Self-study time per day							
	0‐30 min	N/A	N/A	N/A	N/A	N/A	N/A
	31‐60 min	1.87	1.3 to 2.43	<.001	0.87	0.35 to 1.39	.001
	61‐90 min	2.46	1.62 to 3.29	<.001	0.99	0.23 to 1.76	.01
	91 min or more	3.54	2.15 to 4.93	<.001	1.41	0.13 to 2.69	.03
	None	−1.04	−3.29 to 1.21	.37	−0.7	−2.72 to 1.31	.49
Duty hour (h/wk)							
	0‐59	N/A	N/A	N/A	N/A	N/A	N/A
	60‐79	2.27	1.7 to 2.83	<.001	0.97	0.45 to 1.49	<.001
	>80	2.06	1.28 to 2.85	<.001	0.29	−0.45 to 1.04	.44
Using application		2.11	1.59 to 2.63	<.001	0.74	0.25 to 1.22	.003

a GM-ITE: General Medicine In-Training Examination.

b PGY: postgraduate year.

c N/A: not applicable.

## Discussion

### Principal Findings

To our knowledge, this is the first study to compare the characteristics of note-taking app users with those of nonusers among resident physicians actively engaged in clinical practice in Japan. The results showed that app users, compared to nonapp users, tend to be more likely to use online medical resources, have rotated in general medicine, in community-based hospitals, be more experienced trainees, and engage in more self-study. GM-ITE scores were also higher among those who used the app.

Resident physicians who used online resources tended to use the apps more frequently. The use of online resources and apps is considered to be associated with digital literacy. A previous report stated that one of the disadvantages of digital note-taking was a limited period during which students were not able to use the apps easily [21]. Students found apps to be difficult to use if they were using an app that was unfamiliar to them [21]. Another study reported that higher information and digital literacy were associated with satisfactory information retrieval among health care professionals [22]. In a study comparing the effectiveness of information literacy versus digital literacy among family physicians in the Middle East, those with higher information literacy were more effective in searching for information relevant to their practice [23]. Similar to previous reports, both information literacy and digital literacy were involved and affected the frequency of app use.

Participants who used apps tended to have rotated through the Department of General Medicine compared to nonapp users. Japan has an increasingly aging population, and Japanese general medicine doctors are often tasked with treating older adults and are required to deal with multimorbidity [24,25]. Additionally, there have been reports that general medicine doctors based in clinics often use apps to consult with patients during consultations; thus, general practice is highly compatible with app use [26]. Furthermore, as general medicine doctors are keen propagators of education, they could encourage their trainees to use the app, which could have contributed to the results of this study [27].

Our results also indicate that resident physicians in community-based hospitals work in an environment with more app use than in university hospitals. A study of Japanese resident physicians reported that they experienced more cases, spent more time on self-study, worked more hours, and tended to have higher GM-ITE scores in community-based hospitals than university hospitals [28]. Therefore, it is possible that residents in busy community-based hospitals need to compile a large amount of information for self-study, thereby using digital aides to collect and organize this information more efficiently and effectively.

Compared with PGY-1 residents, PGY-2 residents showed a higher rate of app use. There are 2 possible reasons for this. First, PGY-2 residents may have exchanged information with each other on the efficiency of using apps, such as those used for case collection, during their initial year of training and thus have been influenced to use the technology preferentially. In the Japanese training environment, PGY-1s often spend most of their time on mandatory rotations, while PGY-2 students are provided more opportunities for elective rotations. Almost all physicians in Japan proceed to a single specialty department by their PGY-3 year [29]. Therefore, it is possible that PGY-2 residents were motivated to study hard to prepare for their specialty training.

It may be unsurprising that total GM-ITE scores were higher among app users, given that those using digital note-taking have the advantage of being able to organize what they have learned more efficiently than paper note-taking. In the previous studies, high-achieving medical students were aware of the need to manage their time efficiently [30,31]. Increased efficiency is one of the critical concepts of app use. A 2014 report indicated that paper-based systems were more likely to be retained in short-term memory and contribute to higher conceptual awareness; however, a 2022 report showed that short-term memory retention was not significantly different between paper and digital note-taking [32,33]. No reports have compared the facts of paper and digital note-taking on long-term memory; however, digital note-taking may be superior to traditional paper note-taking per efficiency and the amount of information that can be recorded and retained without loss or degradation [11,33]. Thus, the results of this study also support the results of previous reports.

With respect to the GM-ITE score, a positive correlation was observed with being a community-based hospital, self-study time, working hours, the number of emergency shifts, the number of inpatients, and the year of residency, which was consistent with the results of previous reports [27,28]. This suggests that the GM-ITE is a test that is related to clinical practice. The fact that male doctors tended to score higher may have been influenced by the gender hierarchy in Japan [34].

Finally, the app user group spent more time in self-study. App users may have been more motivated, resulting in a more positive effect on clinical performance. Previous studies have also reported that being self-motivated was associated with higher rates of graduation from graduate school [35]. From the results of this study, we consider the possibility that being motivated may have led to an interest in various things, including app use and improved clinical performance.

### Limitations

This study has several limitations. First, our methodology did not specify detailed note-taking procedures, leaving these potentially important specifics unknown. In particular, the survey did not examine the motivation of residents or their ideas about personal efficiency. In this questionnaire survey, the answer item is “30 years old or older”; hence, the exact average age could not be calculated. In addition, this study did not examine the specific product names of the apps, descriptions of data collection methods, frequency of use, satisfaction, or challenges; we wish to investigate these variables in future studies. Also, as this was a cross-sectional study, causal relationships remain unclear. Further, a sample size calculation was not performed because this was an exploratory study. This may have affected the interpretation and effectiveness of the research results.

### Conclusions

This pioneering study on app-based note-taking among physicians revealed that app users are often efficient and motivated, offering valuable insights for medical education. Building on these findings, future research should further explore app-based note-taking and its potential integration into clinical practice.

## Abbreviations:

aD: adjusted difference
aOR: adjusted odds ratio
GM-ITE: General Medicine In-Training Examination
JAMEP: Japan Association for Medical Education Program
MHLW: Ministry of Health, Labor, and Welfare
PGY: postgraduate year
STROBE: Strengthening the Reporting of Observational Studies in Epidemiology
